# A transformer-based genomic prediction method fused with knowledge-guided module

**DOI:** 10.1093/bib/bbad438

**Published:** 2023-12-06

**Authors:** Cuiling Wu, Yiyi Zhang, Zhiwen Ying, Ling Li, Jun Wang, Hui Yu, Mengchen Zhang, Xianzhong Feng, Xinghua Wei, Xiaogang Xu

**Affiliations:** Institute of Intelligent Computing, Zhejiang Lab, Hangzhou 311121, China; Institute of Intelligent Computing, Zhejiang Lab, Hangzhou 311121, China; Institute of Intelligent Computing, Zhejiang Lab, Hangzhou 311121, China; Institute of Intelligent Computing, Zhejiang Lab, Hangzhou 311121, China; Institute of Intelligent Computing, Zhejiang Lab, Hangzhou 311121, China; Northeast Institute of Geography and Agroecology, Chinese Academy of Sciences, Changchun 130012, China; State Key Laboratory of Rice Biology, China National Rice Research Institute, Hangzhou 310006, China; Institute of Intelligent Computing, Zhejiang Lab, Hangzhou 311121, China; Northeast Institute of Geography and Agroecology, Chinese Academy of Sciences, Changchun 130012, China; Institute of Intelligent Computing, Zhejiang Lab, Hangzhou 311121, China; State Key Laboratory of Rice Biology, China National Rice Research Institute, Hangzhou 310006, China; School of Computer and Information Engineering, Zhejiang Gongshang University, Hangzhou 310018, China

**Keywords:** deep learning, Transformer, genomic prediction, knowledge-guided module, prediction method

## Abstract

Genomic prediction (GP) uses single nucleotide polymorphisms (SNPs) to establish associations between markers and phenotypes. Selection of early individuals by genomic estimated breeding value shortens the generation interval and speeds up the breeding process. Recently, methods based on deep learning (DL) have gained great attention in the field of GP. In this study, we explore the application of Transformer-based structures to GP and develop a novel deep-learning model named GPformer. GPformer obtains a global view by gleaning beneficial information from all relevant SNPs regardless of the physical distance between SNPs. Comprehensive experimental results on five different crop datasets show that GPformer outperforms ridge regression-based linear unbiased prediction (RR-BLUP), support vector regression (SVR), light gradient boosting machine (LightGBM) and deep neural network genomic prediction (DNNGP) in terms of mean absolute error, Pearson’s correlation coefficient and the proposed metric consistent index. Furthermore, we introduce a knowledge-guided module (KGM) to extract genome-wide association studies-based information, which is fused into GPformer as prior knowledge. KGM is very flexible and can be plugged into any DL network. Ablation studies of KGM on three datasets illustrate the efficiency of KGM adequately. Moreover, GPformer is robust and stable to hyperparameters and can generalize to each phenotype of every dataset, which is suitable for practical application scenarios.

## INTRODUCTION

Genomic prediction (GP) is a new method for selective breeding using high-density markers covering the entire genome, which can shorten the generation interval and speed up the genetic process [1]. Compared with phenotype selection and marker-assisted selection, GP captures all genetic variation and can conduct genetic evaluation without the need for phenotypic information, thus greatly shortening generation intervals and reducing breeding costs [2–4].

Most existing GP methods are based on best linear unbiased prediction (BLUP) or Bayesian models. According to the difference in statistical models, traditional GP models can generally be divided into two categories: direct methods including GBLUP [5], SSBLUP [6], etc.; indirect methods including ridge regression-based linear unbiased prediction (RR-BLUP) [7] and Bayesian methods such as BayesA, BayesB, BayesC, BayesCπ and Bayes LASSO [1, 8, 9]. Statistical models typically make assumptions about the distribution of effects, which limits the models’ ability to uncover complex relationships between genes and phenotypes. Machine learning (ML) methods do not require prior knowledge of the distribution of variables or the genetic effects of target traits, breaking the restrictions of mixed linear models (MLMs) and Bayesian assumptions [10]. Based on the patterns and trends in the existing datasets, ML methods, such as support vector regression (SVR), random forest and light gradient boosting machine (LightGBM) [11], can improve prediction accuracy through learning and prediction, and perform well in whole-genome selection methods [12–15]. However, ML methods cannot universally improve predictive performance, especially when the number of training samples is far fewer than the number of samples to be predicted [2, 13].

To address the above problems, deep learning (DL) is introduced to the field of GP. Ma *et al.* [16] proposed a deep convolutional neural network called DeepGS to predict phenotypes from genotypes. Liu *et al.* [17] put forward a deep-learning framework DualCNN using a dual stream of convolutional neural networks (CNNs) to predict quantitative traits from single nucleotide polymorphisms (SNPs). Deep neural network genomic prediction (DNNGP) was applied to a variety of omics data to predict phenotypes and achieved better performance compared with other classic models [18].

Until now, most of the DL methods for GP adopt the structure of a CNN [16–18], but the locality of convolutions limits the information flow in the network and makes it difficult to capture the correlation between distant SNPs. Transformer-based models have been proposed to model long-term dependencies for sequential data and reduction of quadratic complexity [19–23] and were also recently applied to regulatory elements prediction or gene expression prediction [24, 25]. Transformers-based models utilize an attention mechanism to handle longer sequences [19]. Nevertheless, gene sequences are much longer than speech sequences or text sequences, making it more challenging to capture the long-term sequences in terms of efficiency and accuracy. Autoformer renovated Transformer into a decomposition forecasting architecture and was used for long-term series forecasting, yielding state-of-the-art accuracy [23]. Based on this intuition, we propose a method called GPformer for GP, which enables efficient sequential-level connection, to expand the effectiveness of information and better aggregate information from SNPs at different distances.

Furthermore, genome-wide association studies (GWAS) and GP are closely related. It is beneficial to use the results of GWAS as prior knowledge to improve GP [26, 27]. So far, to the best of our knowledge, no one has combined GWAS and DL methods for whole-genome prediction. By flexibly and naturally integrated with GWAS, DL models can focus on important loci to better explore the relationship between genes and phenotypes. Meanwhile, if false positives exist in the positions detected by GWAS, DL models can automatically adjust the parameters to achieve the optimal effect. With this idea, we further develop a knowledge-guided module (KGM) based on the results of GWAS and combine it with GPformer to effectively improve the predictive accuracy. Comparisons with other mainstream methods (RR-BLUP, SVR, LightGBM and DNNGP) on different crops such as soybean, rice and wheat show that GPformer and GPformer + KGM are potentially excellent methods for phenotype prediction. The contributions are summarized as follows:

(i) We propose a novel Transformer-based GP method called GPformer, which selectively integrates information from SNPs by capturing long-term dependencies in gene sequences based on an autocorrelation attention mechanism;
(ii) Extensive experiments on five benchmark datasets compared with four mainstream methods adequately illustrate the superiority of GPformer. State-of-the-art performances are reported on the widely used evaluation metrics including mean absolute error (MAE) and Pearson’s correlation coefficient (PCC), as well as the proposed metric CI;
(iii) We develop a flexible KGM based on the results of GWAS and combined it with GPformer to further improve the predictive accuracies. Experimental results demonstrate that GPformer + KGM is superior to other mainstream methods in all-round index evaluation;
(iv) GPformer is robust and stable to hyperparameters. There is no need for GPformer to fine-tune parameters for each phenotype of each dataset, thus being more suitable for practical application scenarios.

## MATERIALS AND METHODS

### Genomic selection datasets

Five datasets are used in this study, including four different crops. The datasets are soybean999, maize282, rice469, wheat599 and wheat2403. Soybean999 is a collection of 999 self-pollinated soybean lines in the F5 generation from the northeast region of China and the dataset is currently not open source. Five consecutive plants of each genotype are selected for data collection at maturity. Phenotypic data are recorded for six agronomic traits including plant height, oil content, protein content, hundred-grain weight, node number and single plant grain weight . The phenotypic values of five consecutive plants are filtered for missing and then averaged. Each plant is genotyped using high-throughput 20 K SNP chip sequencing, resulting in a sequence of 17 847 SNPs. Individuals with a missing rate > 0.1 are eliminated to obtain 999 plants. SNPs with a minor allele frequency < 0.05 and a missing rate > 0.1 are filtered out using PLINK1.9 [28], yielding a total of 7883 SNPs. Then, the missing genotypes are imputed with KNNi imputation by Trait Analysis by Association, Evolution and Linkage (TASSEL) [29].

Maize282 consists of 282 maize association inbred lines, and each line has a sequence of 3093 SNPs. The data are created by the PANZEA project funded by National Science Foundation [30]. Three phenotypes including ear diameter, ear height and days to pollination are considered.

Rice469 is a collection of 469 indica rice varieties from two environments including Hangzhou and Lingshui in China [31]. Each variety is genotyped using high-throughput SNP chip sequencing, resulting in a sequence of 5291 SNPs. Six agronomic traits are considered: plant height, panicle number, panicle length, flag leaf length, tiller number and flag leaf angle.

Wheat599 is from the International Maize and Wheat Improvement Center Global Wheat Program [32], consisting of 599 historical wheat lines genotyped with 1447 DArT (Diversity Array Technology) markers generated by Triticarte Pty. Ltd. (Canberra, Australia; http://www.triticarte.com.au). Markers with allele frequency < 0.05 were removed. Missing genotypes were imputed using samples from the marginal distribution of marker genotypes. A total of 1279 markers are retained after quality control. The phenotype is the average grain yield evaluated in four wheat mega environments.

Wheat2403 consists of 2403 Iranian bread wheat (*Triticum aestivum*) landrace accessions held in CIMMYT’s wheat gene bank [33]. All of the landraces are genotyped using genotyping by sequencing methods, and more than 33 709 SNPs are detected. For each accession, eight phenotypes including thousand-kernel weight, test weight, grain width, grain hardness, grain protein and grain length are considered.

### Evaluation design

In this study, we use 5-fold cross-validation to evaluate the performance of all models. The dataset is randomly divided into 5 folds: one of the 5 folds served as the validation fold and the other 4 folds served as the training folds. This process is repeated 5 times so that each fold is tested once. The performance of each fold is accessed by PCC and the mean average error (MAE) and then averaged over 5 folds. PCC is calculated with the scipy.stats module in Python version 3.9.5. The MAE is defined as follows:


$$ \mathrm{MAE}\left(X,\mathrm{y}\right)=\frac{1}{m}\sum_{i=1}^m\left|f\left({x}_i\right)-{y}_i\right|, $$

where *m* is the number of samples in the test set, $X$ is the genotype input, $y$ is the truth phenotype vector of the test set, $f\left({x}_i\right)$ is the predicted phenotype of the plant $i$ and ${y}_i$ is the truth phenotype of the plant $i$.

**Figure 1:**
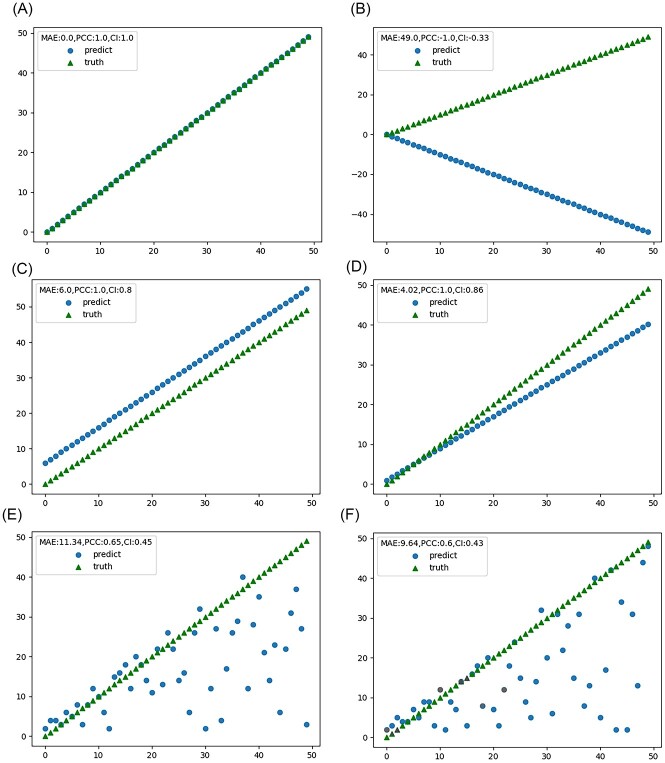
Different MAE and PCC and corresponding CI on simulated data, simulated data with different MAE and same PCC (A,C,D), simulated data with different MAE and different PCC (B,E,F).

PCC indicates whether the trend predicted by the model is accurate, and MAE reflects numerical error. Therefore, we propose a new metric called consistent index (CI) that combines the two to reflect the algorithm’s overall performance. The new metric is defined as follows:


$$ \mathrm{CI}\left(X,y\right)=\frac{\rho_c}{\frac{\mathrm{MAE}\left(X,\mathrm{y}\right)}{\mathrm{mean}\left(|y|\right)}+1}, $$

where ${\rho}_c$ is PCC and $\mathrm{mean}\left(|y|\right)$ is the mean of absolute values of the phenotypes of the test set. The larger the value of CI, the better the performance of the model. The closer the value of CI is to 1, the closer the PCC is to 1 and the closer the MAE is to 0. The term $\mathrm{mean}\left(|y|\right)$ is used as a coefficient to ensure that the value of CI is within a reasonable range. We simulate some data to show that CI can combine PCC and MAE to reflect the overall performance. If multiple models have the same PPC or the same MAE, it is easier to choose one of them (Figure 1A–D). But if both PCC and MAE are different, it is difficult to judge which model is better (Figure 1E and F). Therefore, we combine PCC and MAE as an indicator to reflect model performance and as a criterion for model selection.

### GPformer method

**Figure 2:**
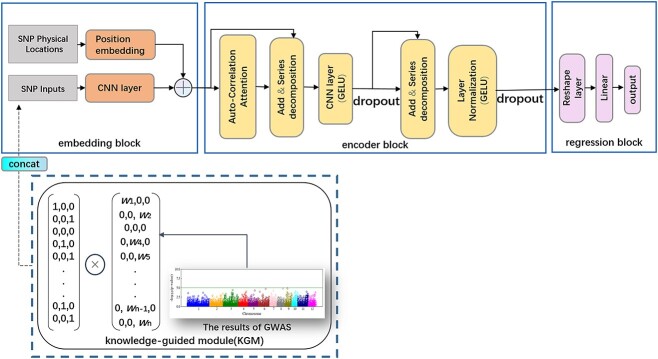
Illustration of model frameworks used in GPformer and KGM.

GPformer consists of three parts: embedding block, encoder block and regression block, where the embedding block is composed of SNP feature embedding and position embedding, the encoder block is composed of one auto-correlation attention layer, two series decomposition layers, one CNN layer, one layer normalization (LayerNorm), two gaussian error linear units (GELU) activation function and two Dropout layers, and the regression block consists of a reshape layer and a linear layer (Figure 2).

The SNP input is coded by sequence or one-hot encoding to obtain the input tensor, then the tensor is input to SNP feature embedding to extract SNP’s features utilizing a convolutional layer. Position embedding takes the physical location information of the SNPs as a input to encode the positional features of the SNPs; if the physical location information does not exist, the sequence number is directly used as the physical location. The positional features extraction method is as follows:


$$ {pe}_t^{(i)}=\left\{\begin{array}{l}\sin \left({\omega}_kt\right), if\ i=2k\\{}\cos \left({\omega}_kt\right), if\ i=2k+1\end{array}\right.\!\!\!\\\!\!\!, $$

where ${p}_{e_t}$is the positional feature at the position *t*, ${pe}_t^{(i)}$ is the $i$th component of the position feature, ${\omega}_k=\frac{1}{10000^{2k/{d}_{\mathrm{model}}}}$,$k=0,1,\dots, \frac{d_{\mathrm{model}}}{2}-1$, ${d}_{\mathrm{model}}$ is the length of the positional feature, *t* is the physical location. The SNP’s features and the positional features are added together for fusion as the input for the encoder block.

The encoder block is the core of the GPformer, which includes the auto-correlation attention layer, series decomposition layer, layer normalization and the GELU activation function. Auto-correlation attention is represented as follows:


$$ {\mathrm{\tau}}_1,\dots, {\mathrm{\tau}}_{\mathrm{k}}={}_{\mathrm{\tau} \in \left\{1,\dots, \mathrm{L}\right\}}{}^{\mathrm{argTopk}}\left({\mathrm{AR}}_{\mathrm{Q},\mathrm{K}}\left(\mathrm{\tau} \right)\right) $$

$$ {\hat{\mathrm{AR}}}_{\mathrm{Q},\mathrm{K}}\left({\mathrm{\tau}}_1\right),\dots, {\hat{\mathrm{AR}}}_{\mathrm{Q},\mathrm{K}}\left({\mathrm{\tau}}_{\mathrm{k}}\right)=\mathrm{SoftMax}\left(\mathrm{A}{\mathrm{R}}_{\mathrm{Q},\mathrm{K}}\left({\mathrm{\tau}}_1\right),\dots, {\mathrm{AR}}_{\mathrm{Q},\mathrm{K}}\left({\mathrm{\tau}}_{\mathrm{k}}\right)\right) $$

$$ \mathrm{AutoCorrelation}\left(\mathrm{Q},\mathrm{K},\mathrm{V}\right)=\sum_{\mathrm{j}=1}^{\mathrm{k}}\mathrm{Delay}\left(\mathrm{V},{\mathrm{\tau}}_{\mathrm{j}}\right){\hat{\mathrm{AR}}}_{\mathrm{Q},\mathrm{K}}\left({\mathrm{\tau}}_{\mathrm{j}}\right) $$

$$ \mathrm{A}{\mathrm{R}}_{\mathrm{Q},\mathrm{K}}\left(\mathrm{\tau} \right)={\mathrm{F}}^{-1}\left(\mathrm{F}\left(\mathrm{Q}\right){\mathrm{F}}^{\ast}\left(\mathrm{K}\right)\right), $$

where $\mathrm{Q},\mathrm{K}\kern0.75em \mathrm{and}\ \mathrm{V}$ is query, key and value, respectively, $\mathrm{argTop}\left(\cdot \right)$ is to get the arguments of the Topk autocorrelations and $\mathrm{k}=\left\lfloor \mathrm{c}\times \mathrm{logL}\right\rfloor$, c is a hyper-parameter. ${\mathrm{AR}}_{\mathrm{Q},\mathrm{K}}$ is autocorrelation between $\mathrm{Q}$ and $\mathrm{K}$. $\mathrm{F}$ denotes Fast Fourier Transforms and ${\mathrm{F}}^{-1}$ is its inverse. ${\mathrm{F}}^{\ast }$ denotes the conjugate operation. $\mathrm{Delay}\left(\mathrm{V},\mathrm{\tau} \right)$ represents the rolling operation to $\mathrm{V}$，and $\mathrm{\tau}$ is the number of places by which the elements of the tensor are shifted. The series decomposition layer is formalized as follows:


$$ {X}_s=X- AvgPool\left( Padding(X)\right), $$

where $X$ is the series features. $AvgPool\left(\cdot \right)$ is the average pooling operation and $Padding\left(\cdot \right)$ is padding operation to keep the series length unchanged.

The activation function in GPformer is GELU, and GELU introduces the idea of random regularization, which is a rough description of neuron input, and is better than ReLU and ELU. This function can be represented as follows:


$$ GELU(x)=x\cdot \frac{1}{2}\left(1+\mathrm{errorfunction}\left(\frac{x}{\sqrt{2}}\right)\right). $$

We can approximate the GELU with


$$ 0.5x\left(1+\mathit{\tanh}\left[\sqrt{\frac{2}{\pi }}\left(x+0.044715{x}^3\right)\right]\right). $$

GPformer uses layer normalization (LayerNorm) to make the input distribution of each layer more stable to help the model converge.

**Figure 3:**
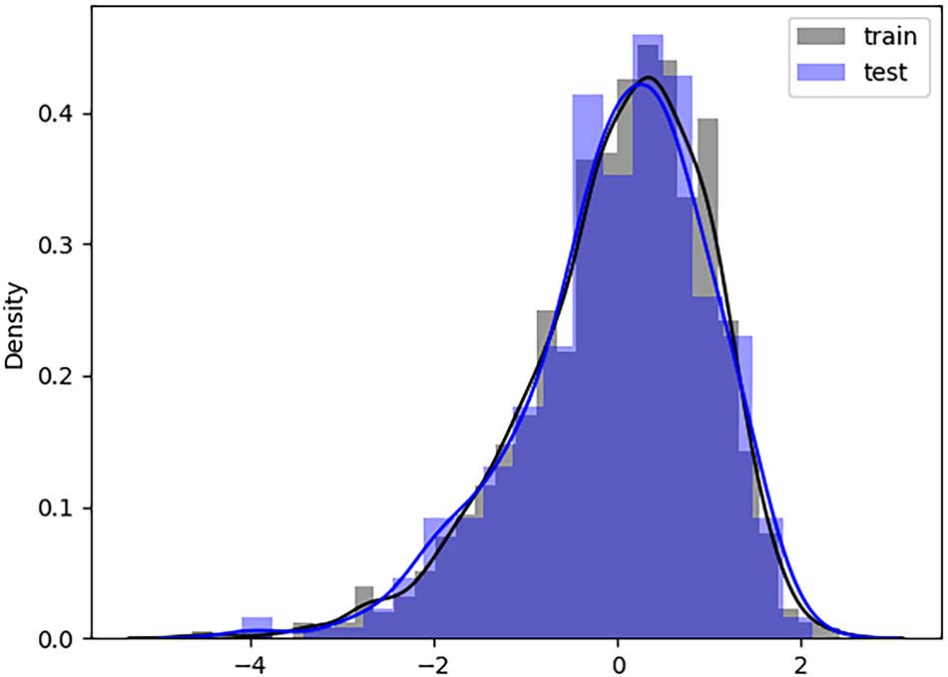
An example of a 1-fold distribution of wheat2403. A bar graph represents a histogram distribution, and the line graph represents the Kolmogorov–Smirnov distribution.

Phenotypic data often exhibit an imbalance phenomenon, whereby the distribution of phenotype data is mostly a normal gaussian distribution (Figure 3): fewer phenotype data at both ends of the distribution and more phenotype data in the middle of the distribution, such data distribution can result in poor model performance on phenotypes with low data volume. Focal loss [34] can solve model performance issues caused by data imbalance in classification, and we transfer the focal loss to the regression problem, and the focal MAE loss is formalized as follows:


$$ \mathrm{loss}=\frac{1}{M}\sum_{i=1}^M{\left(\mathit{\tanh}\left(\beta \ast |{Y}_i-{\mathrm{Y}}_i^{\prime }|\right)\right)}^{\gamma}\ast \mid{Y}_i-{\mathrm{Y}}_i^{\prime}\mid, $$

where *M* denotes the number of individuals in the training batch and $\beta$ and $\gamma$ are hyperparameters. ${Y}_i$ and ${\mathrm{Y}}_i^{\prime }$ are the observed and predicted phenotypic values of the individual $i$, respectively. If individual *i* is in the middle of the normal distribution, due to the large amount of sample data here, the predicted value will be closer to the observed value, and the value of the first term will be small, and vice versa. In this way, the loss function will increase the penalty weight for small sample size and decrease the penalty weight for large sample size. Parameters in the GPformer are optimized with the ADAM [35] optimizer with an initial learning rate of 0.0001 and a weight decay value of 0.00001, and the learning rate drop strategy is implemented using ExponentialLR in Pytorch [36]. The batch size is set to 32 on all datasets. The hyperparameters $\beta$ and $\gamma$ in loss function are 0.5 and 1, respectively. All experiments are implemented in PyTorch and conducted on a single NVIDIA A100 40GB GPU. GPformer is robust and stable, which uses the above parameters for all datasets and all traits.

### The knowledge-guided module

We propose a KGM that can be integrated into any DL model. The main idea of this module is to use biological prior knowledge as an attention mechanism to guide model learning. The biological prior knowledge we use is the results of GWAS. We use the software TASSEL [29] v5.2.81 to do GWAS analysis to obtain *P*-values and *F*-values. The GWAS is performed using three models: general linear model, mixed linear model and compressed linear mixed model. We select one of the three models’ results according to the QQ plot and Manhattan plot. The selection criteria are as follows: firstly, the signal peak is shown in the Manhattan plot, rather than a single isolated point, and secondly, the observed value of the *P-*value in the QQ plot should be located on the diagonal, and the model is unreasonable if it is located below the diagonal. The negative log *P*-values and *F*-values represent the degree of association between genotype and phenotype. If the value is high, it means that it greatly influences the phenotype and vice versa. This can be regarded as an attention mechanism, which means that we can use the GWAS’s results to weigh the input to guide the model to pay different attention to SNPs. The specific method is as follows.

The input genotype data should be encoded using one-hot encoding and then weighted with the negative log *P*-values and *F*-values. The genotypic marker at position *k* is marked as ${pos}_k$, and the encoding is as follows:


$$ \left\{\begin{array}{l}\left(w,0,0\right),\mathrm{if}\ {pos}_k\ \mathrm{is}\ 0/0\\{}\left(0,w,0\right),\mathrm{if}\ {pos}_k\ \mathrm{is}\ 0/1\\{}\left(0,0,w\right),\mathrm{if}\ {pos}_k\ \mathrm{is}\ 1/1\\{}\left(0,0,0\right),\mathrm{if}\ {pos}_k\ \mathrm{is}\ \mathrm{missing}\end{array}\right.. $$

If the *P*-value of ${pos}_k$ is not non-numeric, the value of *w* is $-\log (P)+F\_\mathrm{value}$, otherwise it is 1. The *n* × 3 matrix can be obtained for each given individual after the above encoding, where *n* is the number of genotypic markers. Then, the Hadamard product of the original one-hot encoding and the weighting matrix is performed. The results from the KGM are integrated into the GPformer by concatenating together with the SNP inputs in the embedding block (Figure 2).

## RESULTS

### GPformer outperforms other mainstream methods

**Figure 4:**
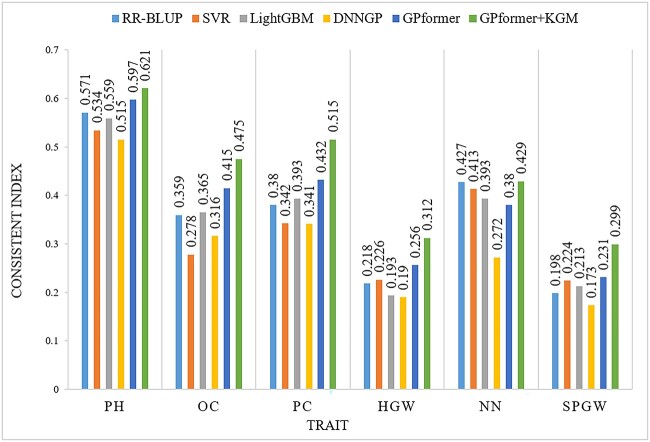
CI of models on the soybean999 dataset. A higher CI indicates a better prediction.

We evaluate RR-BLUP, SVR, LightGBM, DNNGP and GPformer comprehensively in terms of three metrics PCC, MAE and CI. We compare the five methods on different crop datasets, including four small-sized datasets soybean999, maize282, rice469 and wheat599, and one large-sized dataset wheat2403. We first compare all methods on soybean999. On the one hand, according to PCC, GPformer outperforms the other methods in four traits including OC, PC, HGW and SPGW. On the other hand, GPformer is much better than the other methods in all traits evaluated by MAE (Supplementary Table 1, see Supplementary Data available online at http://bib.oxfordjournals.org/). GPformer outperforms the other methods such as RR-BLUP, SVR, LightGBM and DNNGP in five traits by an average of 13.5, 20.7, 14.3 and 28.2% in terms of the new metric CI, respectively (Figure 4 and Supplementary Table 2, see Supplementary Data available online at http://bib.oxfordjournals.org/).

**Figure 5:**
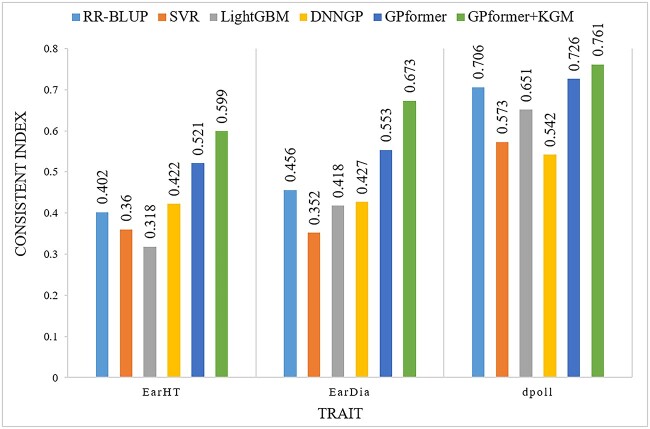
CI of models on the maize282 dataset. A higher CI indicates a better prediction.

We then compare all methods on maize282. The three metrics of GPformer are superior to other methods on all traits. GPformer improves the PCC in three traits by an average of 8.33, 28.98, 21.97 and 18.23% and significantly reduces the MAE in three traits by an average of 78.28, 83.26, 82.96 and 81.68%, respectively, compared with RR-BLUP, SVR, LightGBM and DNNGP (Supplementary Table 3, see Supplementary Data available online at http://bib.oxfordjournals.org/). Overall, GPformer improves the CI in three traits by an average of 8.33, 28.98, 21.97 and 18.23%, respectively, compared with RR-BLUP, SVR, LightGBM and DNNGP (Figure 5 and Supplementary Table 4, see Supplementary Data available online at http://bib.oxfordjournals.org/).

**Figure 6:**
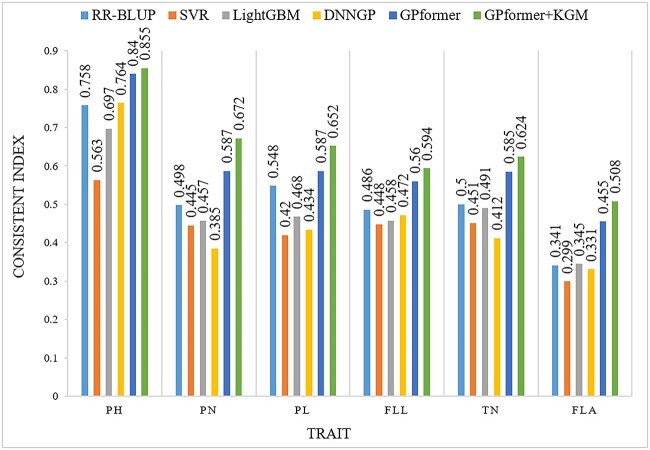
CI of models on the rice469_env1 dataset. A higher CI indicates a better prediction.

**Figure 7:**
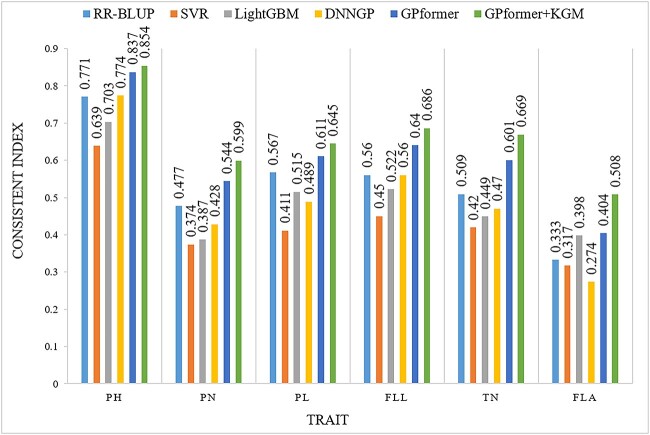
CI of models on the rice469_env2 dataset. A higher CI indicates a better prediction.

The third dataset rice469 is collected from two environments; GPformer performs consistently well in the two environments. In the first environment, GPformer improves the PCC in six traits by an average of 5.87, 20.86, 9.24 and 16.69% and significantly reduces the MAE in six traits by an average of 77.06, 82.09, 82.11 and 81.61%, respectively, compared with RR-BLUP, SVR, LightGBM and DNNGP (Supplementary Table 5, see Supplementary Data available online at http://bib.oxfordjournals.org/). Besides, GPformer improves the CI in six traits by an average of 16.89, 37.98, 24.62 and 32.66%, respectively, compared with RR-BLUP, SVR, LightGBM and DNNGP (Figure 6 and Supplementary Table 6, see Supplementary Data available online at http://bib.oxfordjournals.org/). In the second environment, GPformer improves the PCC in six traits by an average of 3.63, 23.09, 7.56 and 11.45% and significantly reduces the MAE in six traits by an average of 77.45, 82.53, 82.80 and 80.72%, respectively, compared with RR-BLUP, SVR, LightGBM and DNNGP (Supplementary Table 7, see Supplementary Data available online at http://bib.oxfordjournals.org/). Moreover, GPformer improves the CI in six traits by an average of 14, 39.67, 22.67 and 24.99%, respectively, compared with RR-BLUP, SVR, LightGBM and DNNGP (Figure 7 and Supplementary Table 8, see Supplementary Data available online at http://bib.oxfordjournals.org/).

**Figure 8:**
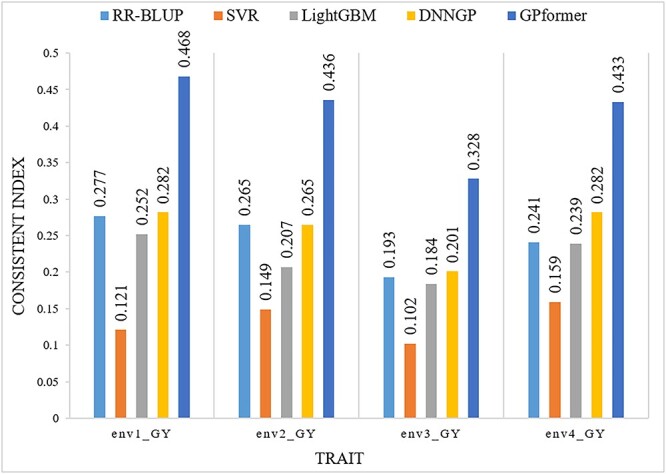
CI of models on the wheat599 dataset. A higher CI indicates a better prediction.

The fourth dataset wheat599 exhibits only one trait from four environments. GPformer improves the PCC in four environments by an average of 10, 92.61, 14.62 and 4.18% and significantly reduces the MAE in four environments by an average of 77.76, 80.46, 80.31 and 77.85%, respectively, compared with RR-BLUP, SVR, LightGBM and DNNGP (Supplementary Table 9, see Supplementary Data available online at http://bib.oxfordjournals.org/). Overall, GPformer improves the CI in four environments by an average of 72.61, 220.97, 90.87 and 63.63%, respectively, compared with RR-BLUP, SVR, LightGBM and DNNGP (Figure 8 and Supplementary Table 10, see Supplementary Data available online at http://bib.oxfordjournals.org/).

**Figure 9:**
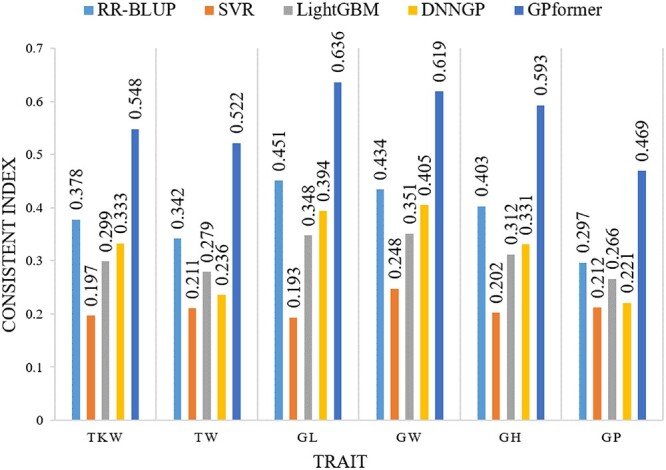
CI of models on the wheat2403 dataset. A higher CI indicates a better prediction.

We further evaluate all methods on the largest size dataset wheat2403 on six traits. GPformer outperforms the other four methods to varying extents in the three metrics. GPformer improves the PCC in six traits by an average of 0.94, 57.4, 6.26 and 18.45% and significantly reduces the MAE in six traits by an average of 75.33, 82.56, 82.66 and 77.72%, respectively, compared with RR-BLUP, SVR, LightGBM and DNNGP (Supplementary Table 11, see Supplementary Data available online at http://bib.oxfordjournals.org/). Furthermore, GPformer improves the CI in six traits by an average of 47.76, 169.98, 82.72 and 82.06%, respectively, compared with RR-BLUP, SVR, LightGBM and DNNGP (Figure 9 and Supplementary Table 12, see Supplementary Data available online at http://bib.oxfordjournals.org/).

To sum up, the results indicate that RR-BLUP is relatively stable and robust on datasets of different sizes but performs badly in terms of MAE. SVR and LightGBM are not consistent and stable on all traits, especially LightGBM performs not well on small-size datasets. DNNGP performs badly when the number of SNP sites is large and is not robust because it requires fine-tuning of parameters for each trait in each dataset. GPformer has more stable and encouraging performance on all datasets in terms of the three metrics. It depends on the use of the Transformer-based structure to mine the relationship between long-distance loci and the genotype–phenotype relationship, as well as the focal loss to alleviate sample imbalance.

### Prediction accuracy of GPformer + KGM

GWAS and genome prediction are closely related because they are both studied using genome-wide markers. The results of GWAS can be viewed as prior knowledge, which can be incorporated into DL models as an attention mechanism. Specifically, we propose a module called the KGM, which uses the sum of negative logarithmic *P*-values and *F*-values in GWAS results as attention weighting for gene inputs. We compare the GPformer + KGM method with others on three datasets: soybean999, maize282 and rice469.

On the soybean999 dataset, GPformer + KGM has further improved compared with other methods, and it outperforms the other methods such as RR-BLUP, SVR, LightGBM and DNNGP in six traits by an average of 20.89, 30.03, 25 and 38.45% in the metric PCC and 28.47, 35.45, 30.56 and 52.55% in the metric CI (Figure 4 and Supplementary Tables 1 and 2, see Supplementary Data available online at http://bib.oxfordjournals.org/).

On the maize282 dataset, GPformer + KGM has the largest improvement of 88.32% compared with other methods (Figure 5 and Supplementary Tables 3 and 4, see Supplementary Data available online at http://bib.oxfordjournals.org/).

On the rice469 dataset, in the first environment, GPformer + KGM improves the PCC in six traits by an average of 14.92, 31.14, 18.66, 26.89 and 8.5% and significantly reduces the MAE in six traits by an average of 77.82, 82.76, 82.79, 82.07 and 3.12%, respectively, compared with RR-BLUP, SVR, LightGBM, DNNGP and GPformer. In addition, GPformer + KGM improves the CI in six traits by an average of 27.09, 49.84, 35.51, 44.61 and 8.62%, respectively, compared with RR-BLUP, SVR, LightGBM, DNNGP and GPformer (Figure 6 and Supplementary Tables 5 and 6, see Supplementary Data available online at http://bib.oxfordjournals.org/). In the second environment, GPformer + KGM improves the PCC in six traits by an average of 14.26, 35.31, 18.06, 23.34 and 10.16% and significantly reduces the MAE in six traits by an average of 78.46, 83.34, 83.58, 81.59 and 4.5%, respectively, compared with RR-BLUP, SVR, LightGBM, DNNGP and GPformer. Overall, GPformer + KGM improves the CI in six traits by an average of 26.06, 53.81, 34.89, 38.75 and 10.31% compared with RR-BLUP, SVR, LightGBM, DNNGP and GPformer (Figure 7 and Supplementary Tables 7 and 8, see Supplementary Data available online at http://bib.oxfordjournals.org/). The results suggest that GPformer + KGM is superior to other mainstream methods in all-around index evaluation.

## DISCUSSION

In this study, we explore the application of Transformer-based structures in the field of GP and develop a model called GPformer. GPformer consists of three parts: embedding block, encoder block and regression block. Genomic data are naturally sequential and has positional information. The embedding block is adept at handling sequence input and can fuse gene sequence features and position information. The encoder block extracts associations between different gene loci to construct nonlinear relationships between genes and phenotypes, and the core of which is an auto-correlation mechanism to achieve efficient sequence-level connections to extend the utility of information. The auto-correlation mechanism is designed especially for dependencies discovery and aggregation of information at the series level. The weights of auto-attention depend on the aggregation of similar sub-series in the underlying series, which allows the model to improve predictions by collecting information on all related SNPs regardless of the relationship between SNPs. This results in a better flow of information between loci since each position is directly concerned with all other positions in the sequence. The regression block consists of a reshape layer and a linear layer for feature aggregation and regression prediction.

GPformer can greatly expand the receptive field and increase the information flow. The receptive field of CNN is limited by the size of the convolution kernel, so the modeling range of CNN can only be within the receptive field of a convolution kernel; the Transformer-based structures use attention mechanism to model global information, so the perceptual range is the entire input [37].

Furthermore, we introduce an improved focal loss to address the data imbalance problem, which may cause the network to be more biased toward learning more data-dominated classes and degrade the model performance. Besides, we recommend the new metric CI which combining MAE and PCC to comprehensively evaluate model performance.

We also investigate the combination of biological prior knowledge and DL. Previous methods combine the results of GWAS as fixed effects with traditional statistical models [26, 27]. These methods are difficult to eliminate the misleading of false positives and only use some highly significant loci. We try to weigh the gene input with the results of GWAS to guide the model to pay different attention to SNPs to further improve performance. As a result, we develop a KGM based on the results of GWAS and combined it with GPformer to effectively improve the predictive accuracies. The experimental results of GPformer + KGM on three datasets show that GWAS is a powerful assistant when combined with DL. Using GWAS to estimate marker importance value or screen the SNPs that are significantly related to traits could increase the trait predictability in GS [38, 39]. A small *P*-value indicates that the target SNP is related to the phenotype and vice versa, which can be considered as prior knowledge or attention mechanism. By flexibly and naturally integrated with GWAS, DL models can focus on loci of high significance and less on loci of low significance, thereby reducing the difficulty of modeling and better exploring the relationship between genes and phenotypes. Meanwhile, if false positives exist in the positions detected by GWAS, DL models can automatically adjust the parameters to achieve optimal performance. In future work, we will investigate the combination of other biological priors with DL and explore new ways of fusion to make AI + breeding more applicable to practical breeding.

**Key Points**

- We propose a novel Transformer-based genomic prediction method called GPformer.
- We develop a flexible knowledge-guided module (KGM) based on the results of GWAS and combined it with GPformer to further improve the predictive accuracies.
- Extensive experiments adequately illustrate the superiority of GPformer and KGM.
- GPformer is robust and stable to hyperparameters.

## Supplementary Material

supplementary_bib_bbad438

## Data Availability

The data that support the findings of this study are available from the corresponding author upon reasonable request.
