# Supplementary material for: A transformer-based genomic prediction method fused with knowledge-guided module
Source: Brief Bioinform. 2023 Dec 6;25(1):bbad438. doi: 10.1093/bib/bbad438 (PMC10701102; doi:10.1093/bib/bbad438)
Supplement: supplementary_bib_bbad438 [file supplementary_bib_bbad438.docx]

**A Transformer-based Genomic Prediction Method fused with Knowledge-guided Module**

Cuiling Wu^1^, Yiyi Zhang^1^, Zhiwen Ying^1^, Ling Li^1^, Jun Wang^1^, Hui Yu^2^, Mengchen Zhang^3^, Xianzhong Feng^12^, Xinghua Wei^13^, Xiaogang Xu^4^*

^1^ Institute of Intelligent Computing, Zhejiang Lab, Hangzhou 311121, China

^2^ Northeast Institute of Geography and Agroecology, Chinese Academy of Sciences, Changchun 130012, China

^3^ State Key Laboratory of Rice Biology, China National Rice Research Institute, Hangzhou 310006, China

^4^ School of Computer and Information Engineering, Zhejiang Gongshang University, Hangzhou 310018, China

* Corresponding author:xxgang2013@163.com

**Supporting information**

**Supplemental Table 1. PCC and MAE of six models on the soybean999 dataset. A higher PCC and a lower MAE indicate a better prediction.**

| Models | Metric | PH | OC | PC | HGW | NN | SPGW |
| --- | --- | --- | --- | --- | --- | --- | --- |
| RR-BLUP | PCC | 0.616 | 0.391 | 0.39 | 0.234 | 0.449 | 0.237 |
|  | MAE | 8.19 | 1.81 | 1.08 | 1.57 | 0.92 | 3.76 |
| SVR | PCC | 0.586 | 0.293 | 0.36 | 0.242 | 0.43 | 0.242 |
|  | MAE | 10.1 | 1.11 | 2.02 | 1.52 | 0.75 | 1.52 |
| LightGBM | PCC | 0.613 | 0.386 | 0.413 | 0.207 | 0.411 | 0.231 |
|  | MAE | 9.97 | 1.13 | 2.02 | 1.58 | 0.86 | 1.64 |
| DNNGP | PCC | 0.562 | 0.364 | 0.38 | 0.211 | 0.294 | 0.21 |
|  | MAE | 9.34 | 3.05 | 4.54 | 2.33 | 1.47 | 4.07 |
| **GPformer** | PCC | **0.609** | **0.42** | **0.436** | **0.261** | **0.384** | **0.245** |
|  | MAE | **2.15** | **0.24** | **0.38** | **0.4** | **0.22** | **1.21** |
| **GPformer + KGM** | PCC | **0.633** | **0.483** | **0.525** | **0.318** | **0.435** | **0.312** |
|  | MAE | **2.05** | **0.32** | **0.79** | **0.44** | **0.25** | **0.85** |

Note: PH, plant height; OC, oil content; PC, protein content; HGW, hundred-grain weight; NN, node number; SPGW, single plant grain weight; RR-BLUP, ridge regression-based linear unbiased prediction; SVR, support vector regression; LightGBM, light gradient boosting machine; DNNGP, deep neural network for genomic prediction. GPformer, A Transformer-based method for genomic prediction; GPformer + KGM, A Transformer-based method for genomic prediction with a knowledge-guided module.

**Supplemental Table 2. CI of six models on the soybean999 dataset. A higher CI indicates a better prediction.**

| Models | PH | OC | PC | HGW | NN | SPGW |
| --- | --- | --- | --- | --- | --- | --- |
| RR-BLUP | 0.571 | 0.359 | 0.380 | 0.218 | 0.427 | 0.198 |
| SVR | 0.534 | 0.278 | 0.342 | 0.226 | 0.413 | 0.224 |
| LightGBM | 0.559 | 0.365 | 0.393 | 0.193 | 0.393 | 0.213 |
| DNNGP | 0.515 | 0.316 | 0.341 | 0.190 | 0.272 | 0.173 |
| **GPformer** | **0.597** | **0.415** | **0.432** | **0.256** | **0.380** | **0.231** |
| **GPformer + KGM** | **0.621** | **0.475** | **0.515** | **0.312** | **0.429** | **0.299** |

Note: PH, plant height; OC, oil content; PC, protein content; HGW, hundred-grain weight; NN, node number; SPGW, single plant grain weight; RR-BLUP, ridge regression-based linear unbiased prediction; SVR, support vector regression; LightGBM, light gradient boosting machine; DNNGP, deep neural network for genomic prediction. GPformer, A Transformer-based method for genomic prediction; GPformer + KGM, A Transformer-based method for genomic prediction with a knowledge-guided module.

**Supplemental Table 3. PCC and MAE of six models on the maize282 dataset. A higher PCC and a lower MAE indicate a better prediction.**

| Models | Metric | EarHT | EarDia | dpoll |
| --- | --- | --- | --- | --- |
| RR-BLUP | PCC | 0.49 | 0.491 | 0.738 |
|  | MAE | 13.5 | 2.84 | 3.04 |
| SVR | PCC | 0.45 | 0.385 | 0.612 |
|  | MAE | 15.33 | 3.43 | 4.61 |
| LightGBM | PCC | 0.398 | 0.454 | 0.696 |
|  | MAE | 15.41 | 3.17 | 4.71 |
| DNNGP | PCC | 0.513 | 0.463 | 0.578 |
|  | MAE | 13.21 | 3.13 | 4.45 |
| **Gpformer** | PCC | **0.541** | **0.562** | **0.739** |
|  | MAE | **2.36** | **0.57** | **0.84** |
| **GPformer + KGM** | PCC | **0.62** | **0.682** | **0.77** |
|  | MAE | **2.12** | **0.49** | **0.82** |

Note: EarDia, ear diameter; EarHT, ear height; dpoll, days to pollination; RR-BLUP, ridge regression-based linear unbiased prediction; SVR, support vector regression; LightGBM, light gradient boosting machine; DNNGP, deep neural network for genomic prediction. GPformer, A Transformer-based method for genomic prediction; GPformer + KGM, A Transformer-based method for genomic prediction with a knowledge-guided module.

**Supplemental Table 4. CI of six models on the maize282 dataset. A higher CI indicates a better prediction.**

| Models | EarHT | EarDia | dpoll |
| --- | --- | --- | --- |
| RR-BLUP | 0.402 | 0.456 | 0.706 |
| SVR | 0.360 | 0.352 | 0.573 |
| LightGBM | 0.318 | 0.418 | 0.651 |
| DNNGP | 0.422 | 0.427 | 0.542 |
| **GPformer** | **0.521** | **0.553** | **0.726** |
| **GPformer + KGM** | **0.599** | **0.673** | **0.761** |

Note: EarDia, ear diameter; EarHT, ear height; dpoll, days to pollination; RR-BLUP, ridge regression-based linear unbiased prediction; SVR, support vector regression; LightGBM, light gradient boosting machine; DNNGP, deep neural network for genomic prediction. GPformer, A Transformer-based method for genomic prediction; GPformer + KGM, A Transformer-based method for genomic prediction with a knowledge-guided module.

**Supplemental Table 5. PCC and MAE of six models on the rice469 dataset in environment 1. A higher PCC and a lower MAE indicate a better prediction.**

| Models | Metric | PH | PN | PL | FLL | TN | FLA |
| --- | --- | --- | --- | --- | --- | --- | --- |
| RR-BLUP | PCC | 0.831 | 0.58 | 0.583 | 0.538 | 0.583 | 0.422 |
|  | MAE | 10.64 | 1.52 | 1.41 | 2.92 | 2.07 | 4.35 |
| SVR | PCC | 0.672 | 0.535 | 0.452 | 0.507 | 0.543 | 0.379 |
|  | MAE | 21.52 | 1.89 | 1.72 | 3.61 | 2.53 | 4.86 |
| LightGBM | PCC | 0.831 | 0.548 | 0.504 | 0.519 | 0.591 | 0.439 |
|  | MAE | 21.23 | 1.86 | 1.71 | 3.62 | 2.55 | 4.97 |
| DNNGP | PCC | 0.842 | 0.475 | 0.493 | 0.53 | 0.488 | 0.413 |
|  | MAE | 11.34 | 2.18 | 3.05 | 3.33 | 2.31 | 4.52 |
| **GPformer** | PCC | **0.856** | **0.609** | **0.596** | **0.574** | **0.608** | **0.481** |
|  | MAE | **2.14** | **0.35** | **0.34** | **0.66** | **0.49** | **1.05** |
| **GPformer + KGM** | PCC | **0.873** | **0.694** | **0.661** | **0.608** | **0.648** | **0.537** |
|  | MAE | **2.27** | **0.3** | **0.32** | **0.65** | **0.47** | **1.06** |

Note: PH, plant height; PN, panicle number; PL, panicle length; FLL, flag leaf length; TN, tiller number; FLA, flag leaf angle; RR-BLUP, ridge regression-based linear unbiased prediction; SVR, support vector regression; LightGBM, light gradient boosting machine; DNNGP, deep neural network for genomic prediction. GPformer, A Transformer-based method for genomic prediction; GPformer + KGM, A Transformer-based method for genomic prediction with a knowledge-guided module.

**Supplemental Table 6. CI of six models on the rice469 dataset in environment 1. A higher CI indicates a better prediction.**

| Models | PH | PN | PL | FLL | TN | FLA |
| --- | --- | --- | --- | --- | --- | --- |
| RR-BLUP | 0.758 | 0.498 | 0.548 | 0.486 | 0.500 | 0.341 |
| SVR | 0.563 | 0.445 | 0.420 | 0.448 | 0.451 | 0.299 |
| LightGBM | 0.697 | 0.457 | 0.468 | 0.458 | 0.491 | 0.345 |
| DNNGP | 0.764 | 0.385 | 0.434 | 0.472 | 0.412 | 0.331 |
| **GPformer** | **0.840** | **0.587** | **0.587** | **0.560** | **0.585** | **0.455** |
| **GPformer + KGM** | **0.855** | **0.672** | **0.652** | **0.594** | **0.624** | **0.508** |

Note: PH, plant height; PN, panicle number; PL, panicle length; FLL, flag leaf length; TN, tiller number; FLA, flag leaf angle; RR-BLUP, ridge regression-based linear unbiased prediction; SVR, support vector regression; LightGBM, light gradient boosting machine; DNNGP, deep neural network for genomic prediction. GPformer, A Transformer-based method for genomic prediction; GPformer + KGM, A Transformer-based method for genomic prediction with a knowledge-guided module.

**Supplemental Table 7. PCC and MAE of six models on the rice469 dataset in environment 2. A higher PCC and a lower MAE indicate a better prediction.**

| Models | Metric | PH | PN | PL | FLL | TN | FLA |
| --- | --- | --- | --- | --- | --- | --- | --- |
| RR-BLUP | PCC | 0.846 | 0.554 | 0.601 | 0.623 | 0.604 | 0.393 |
|  | MAE | 13.09 | 1.48 | 1.56 | 4.38 | 2.43 | 2.94 |
| SVR | PCC | 0.766 | 0.447 | 0.442 | 0.517 | 0.512 | 0.377 |
|  | MAE | 26.77 | 1.77 | 1.98 | 5.85 | 2.88 | 3.09 |
| LightGBM | PCC | 0.844 | 0.463 | 0.554 | 0.599 | 0.559 | 0.473 |
|  | MAE | 26.91 | 1.79 | 1.95 | 5.74 | 3.21 | 3.08 |
| DNNGP | PCC | 0.853 | 0.519 | 0.53 | 0.627 | 0.572 | 0.326 |
|  | MAE | 13.67 | 1.94 | 2.19 | 4.68 | 2.85 | 3.11 |
| **GPformer** | PCC | **0.855** | **0.565** | **0.62** | **0.655** | **0.627** | **0.419** |
|  | MAE | **2.92** | **0.35** | **0.39** | **0.93** | **0.56** | **0.59** |
| **GPformer + KGM** | PCC | **0.872** | **0.623** | **0.654** | **0.702** | **0.695** | **0.525** |
|  | MAE | **2.79** | **0.35** | **0.37** | **0.93** | **0.51** | **0.54** |

Note: PH, plant height; PN, panicle number; PL, panicle length; FLL, flag leaf length; TN, tiller number; FLA, flag leaf angle; RR-BLUP, ridge regression-based linear unbiased prediction; SVR, support vector regression; LightGBM, light gradient boosting machine; DNNGP, deep neural network for genomic prediction. GPformer, A Transformer-based method for genomic prediction; GPformer + KGM, A Transformer-based method for genomic prediction with a knowledge-guided module.

**Supplemental Table 8. CI of six models on the rice469 dataset in environment 2. A higher CI indicates a better prediction.**

| Models | PH | PN | PL | FLL | TN | FLA |
| --- | --- | --- | --- | --- | --- | --- |
| RR-BLUP | 0.771 | 0.477 | 0.567 | 0.560 | 0.509 | 0.333 |
| SVR | 0.639 | 0.374 | 0.411 | 0.450 | 0.420 | 0.317 |
| LightGBM | 0.703 | 0.387 | 0.515 | 0.522 | 0.449 | 0.398 |
| DNNGP | 0.774 | 0.428 | 0.489 | 0.560 | 0.470 | 0.274 |
| **GPformer** | **0.837** | **0.544** | **0.611** | **0.640** | **0.601** | **0.404** |
| **GPformer + KGM** | **0.854** | **0.599** | **0.645** | **0.686** | **0.669** | **0.508** |

Note: PH, plant height; PN, panicle number; PL, panicle length; FLL, flag leaf length; TN, tiller number; FLA, flag leaf angle; RR-BLUP, ridge regression-based linear unbiased prediction; SVR, support vector regression; LightGBM, light gradient boosting machine; DNNGP, deep neural network for genomic prediction. GPformer, A Transformer-based method for genomic prediction; GPformer + KGM, A Transformer-based method for genomic prediction with a knowledge-guided module.

**Supplemental Table 9. PCC and MAE of six models on the wheat599 dataset. A higher PCC and a lower MAE indicate a better prediction.**

| Models | Metric | env1_GY | env2_GY | env3_GY | env4_GY |
| --- | --- | --- | --- | --- | --- |
| RR-BLUP | PCC | 0.508 | 0.491 | 0.37 | 0.455 |
|  | MAE | 0.67 | 0.66 | 0.72 | 0.69 |
| SVR | PCC | 0.237 | 0.299 | 0.206 | 0.317 |
|  | MAE | 0.77 | 0.78 | 0.8 | 0.77 |
| LightGBM | PCC | 0.505 | 0.414 | 0.361 | 0.473 |
|  | MAE | 0.81 | 0.77 | 0.76 | 0.76 |
| DNNGP | PCC | 0.523 | 0.497 | 0.384 | 0.522 |
|  | MAE | 0.69 | 0.68 | 0.72 | 0.66 |
| **GPformer** | PCC | **0.561** | **0.515** | **0.399** | **0.532** |
|  | MAE | **0.16** | **0.14** | **0.17** | **0.14** |

Note: GY, grain yield; RR-BLUP, ridge regression-based linear unbiased prediction; SVR, support vector regression; LightGBM, light gradient boosting machine; DNNGP, deep neural network for genomic prediction. GPformer, A Transformer-based method for genomic prediction.

**Supplemental Table 10. CI of six models on the wheat599 dataset. A higher CI indicates a better prediction.**

| Models | env1_GY | env2_GY | env3_GY | env4_GY |
| --- | --- | --- | --- | --- |
| RR-BLUP | 0.277 | 0.265 | 0.193 | 0.241 |
| SVR | 0.121 | 0.149 | 0.102 | 0.159 |
| LightGBM | 0.252 | 0.207 | 0.184 | 0.239 |
| DNNGP | 0.282 | 0.265 | 0.201 | 0.282 |
| **GPformer** | **0.468** | **0.436** | **0.328** | **0.433** |

Note: GY, grain yield; RR-BLUP, ridge regression-based linear unbiased prediction; SVR, support vector regression; LightGBM, light gradient boosting machine; DNNGP, deep neural network for genomic prediction. GPformer, A Transformer-based method for genomic prediction.

**Supplemental Table 11. PCC and MAE of six models on the wheat2403 dataset. A higher PCC and a lower MAE indicate a better prediction.**

| Models | Metric | TKW | TW | GL | GW | GH | GP |
| --- | --- | --- | --- | --- | --- | --- | --- |
| RR-BLUP | PCC | 0.657 | 0.609 | 0.741 | 0.725 | 0.684 | 0.54 |
|  | MAE | 0.6 | 0.61 | 0.5 | 0.53 | 0.57 | 0.65 |
| SVR | PCC | 0.401 | 0.4313 | 0.389 | 0.501 | 0.405 | 0.424 |
|  | MAE | 0.84 | 0.82 | 0.79 | 0.81 | 0.82 | 0.79 |
| LightGBM | PCC | 0.626 | 0.571 | 0.706 | 0.702 | 0.621 | 0.531 |
|  | MAE | 0.89 | 0.82 | 0.8 | 0.79 | 0.81 | 0.79 |
| DNNGP | PCC | 0.595 | 0.446 | 0.683 | 0.692 | 0.593 | 0.422 |
|  | MAE | 0.64 | 0.7 | 0.57 | 0.56 | 0.65 | 0.72 |
| **GPformer** | PCC | **0.658** | **0.622** | **0.742** | **0.729** | **0.687** | **0.552** |
|  | MAE | **0.16** | **0.15** | **0.13** | **0.14** | **0.13** | **0.14** |

Note: TKW, thousand-kernel weight; TW, test weight; GW, grain width; GH, grain hardness; GP, grain protein; GL, grain length; RR-BLUP, ridge regression-based linear unbiased prediction; SVR, support vector regression; LightGBM, light gradient boosting machine; DNNGP, deep neural network for genomic prediction. GPformer, A Transformer-based method for genomic prediction.

**Supplemental Table 12. CI of six models on the wheat wheat2403 dataset. A higher CI indicates a better prediction.**

| Models | TKW | TW | GL | GW | GH | GP |
| --- | --- | --- | --- | --- | --- | --- |
| RR-BLUP | 0.378 | 0.342 | 0.451 | 0.434 | 0.403 | 0.297 |
| SVR | 0.197 | 0.211 | 0.193 | 0.248 | 0.202 | 0.212 |
| LightGBM | 0.299 | 0.279 | 0.348 | 0.351 | 0.312 | 0.266 |
| DNNGP | 0.333 | 0.236 | 0.394 | 0.405 | 0.331 | 0.221 |
| **GPformer** | **0.548** | **0.522** | **0.636** | **0.619** | **0.593** | **0.469** |

Note: TKW, thousand-kernel weight; TW, test weight; GW, grain width; GH, grain hardness; GP, grain protein; GL, grain length; RR-BLUP, ridge regression-based linear unbiased prediction; SVR, support vector regression; LightGBM, light gradient boosting machine; DNNGP, deep neural network for genomic prediction. GPformer, A Transformer-based method for genomic prediction.

**Supplemental Table 13. Parameters used by different models.**

|  | SVR | LightGBM | | | |
| --- | --- | --- | --- | --- | --- |
| Datasets | kernel | objective | num_leaves | learning_rate | n_estimators |
| soybean999 | linear | regression | 15 | 0.1 | 20 |
| maize282 | linear | regression | 15 | 0.1 | 20 |
| rice469_env1 | linear | regression | 15 | 0.1 | 20 |
| rice469_env2 | linear | regression | 15 | 0.1 | 20 |
| wheat599 | linear | regression | 15 | 0.1 | 20 |
| wheat2403 | linear | regression | 15 | 0.1 | 20 |

| DNNGP | | | | | | | | |
| --- | --- | --- | --- | --- | --- | --- | --- | --- |
| Datasets | Traits | | learning_rate | | batch size | epoch | | dropout |
| soybean999 | all traits | | 0.001 | | 23 | 100 | | 0.3 |
| maize282 | all traits | | 0.001 | | 23 | 100 | | 0.3 |
| rice469_env1 | all traits | | 0.001 | | 23 | 100 | | 0.3 |
| rice469_env2 | all traits | | 0.001 | | 23 | 100 | | 0.3 |
| wheat599 | env1_GY | | 0.001 | | 41 | 100 | | 0.3 |
|  | env2_GY | | 0.005 | | 32 | 100 | | 0.3 |
|  | env3_GY | | 0.005 | | 23 | 100 | | 0.3 |
|  | env4_GY | | 0.001 | | 18 | 100 | | 0.3 |
| wheat2403 | TKW | | 0.001 | | 14 | 100 | | 0.3 |
|  | TW | | 0.005 | | 14 | 100 | | 0.3 |
|  | GL | | 0.001 | | 17 | 100 | | 0.3 |
|  | GW | | 0.001 | | 17 | 100 | | 0.3 |
|  | GH | | 0.01 | | 12 | 100 | | 0.3 |
|  | GP | | 0.001 | | 10 | 100 | | 0.3 |
| GPformer/GPformer + KGM | | | | | | | | |
| Datasets | learning_rate | batch size | | epoch | | | dropout | |
| soybean999 | 0.0001 | 32 | | 100 | | | 0.1 | |
| maize282 | 0.0001 | 32 | | 100 | | | 0.1 | |
| rice469_env1 | 0.0001 | 32 | | 100 | | | 0.1 | |
| rice469_env2 | 0.0001 | 32 | | 100 | | | 0.1 | |
| wheat599 | 0.0001 | 32 | | 100 | | | 0.1 | |
| wheat2403 | 0.0001 | 32 | | 100 | | | 0.1 | |

Note: Unsubdivided cells represent all phenotypes with the same parameters
